# Identifying Suspected ATTR-CM From Unstructured EHR Data Using AI

**DOI:** 10.1016/j.jacadv.2026.103245

**Published:** 2026-10-21

**Authors:** Bruno Batinica, Richard K. Cheng, Rohan Khera

**Affiliations:** a Section of Cardiovascular Medicine, Department of Internal Medicine, Yale School of Medicine, New Haven, Connecticut, USA; b Cardiovascular Data Science (CarDS) Lab, Yale School of Medicine, New Haven, Connecticut, USA; c Division of Cardiology, University of Washington Medical Center, Seattle, Washington, USA; d Section of Health Informatics, Department of Biostatistics, Yale School of Public Health, New Haven, Connecticut, USA; e Section of Biomedical Informatics and Data Science, Yale School of Medicine, New Haven, Connecticut, USA; f Center for Outcomes Research and Evaluation, Yale-New Haven Hospital, New Haven, Connecticut, USA

**Keywords:** artificial intelligence, natural language processing, transthyretin amyloid cardiomyopathy

**Figure:**
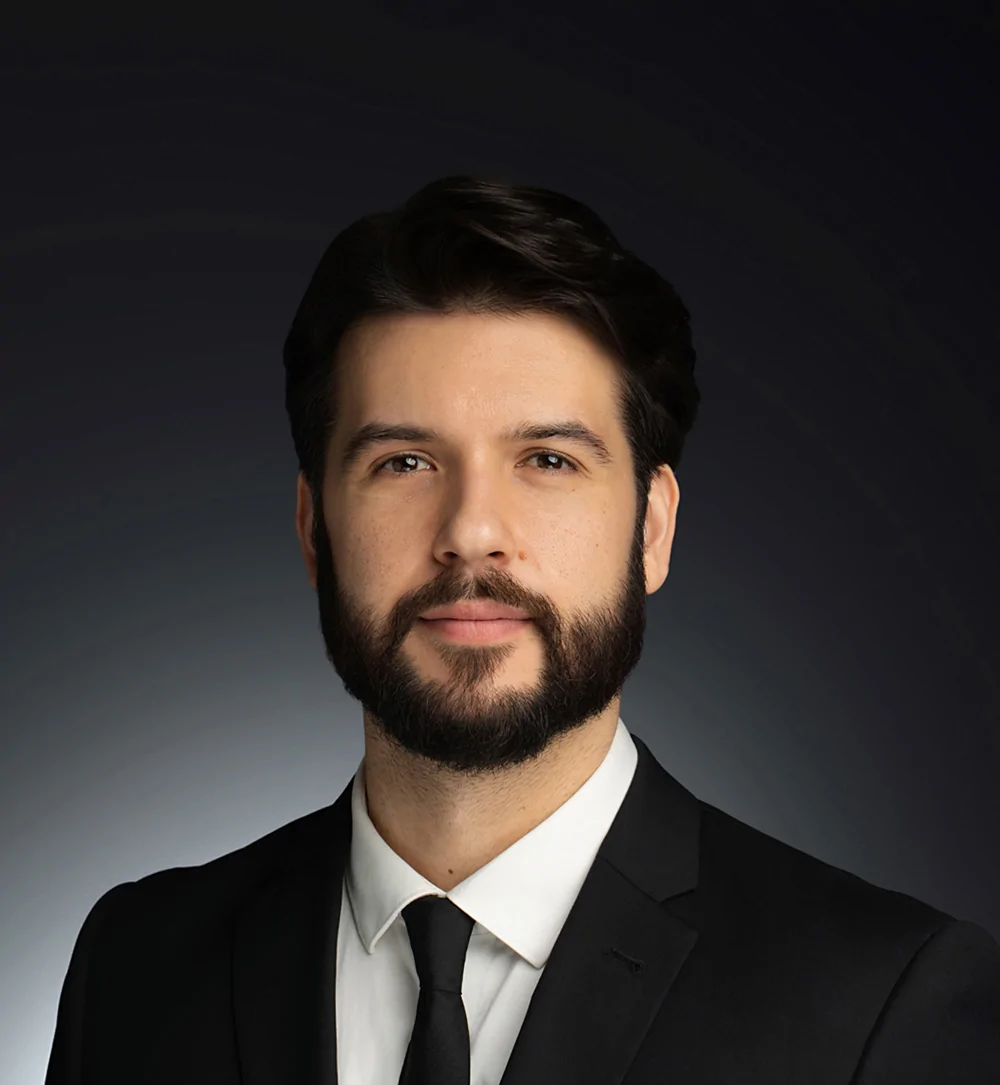

Transthyretin amyloid cardiomyopathy (ATTR-CM) is a progressive infiltrative disease with an untreated median survival of under 5 years.^1^ Patients frequently present with nonspecific symptoms across multiple subspecialties before the diagnosis is made, and an unknown proportion are never diagnosed. Effective therapies now exist that produce the greatest benefit when initiated early, and confirmatory testing is typically noninvasive, thus presenting a critical opportunity for disease screening. Artificial intelligence approaches to ATTR-CM screening have largely concentrated on using structured information or physiological signals (eg, electrocardiograms and echocardiograms) as inputs.^2^, ^3^, ^4^, ^5^ However, pertinent information regarding ATTR-CM may reside in unstructured clinical and imaging notes, where extracardiac manifestations, qualitative imaging descriptions, and clinical suspicion itself are recorded. Advances in natural language processing (NLP) have made the extraction of such content from large record corpora tractable, handling negation and context that constrained earlier rule-based approaches, and comparable methods have been applied successfully in heart failure phenotyping.^6^^,^^7^ In this issue of *JACC: Advances*, the study by Arora et al^8^ demonstrates an important application of NLP in identifying patterns suggestive of suspected ATTR-CM in clinical and imaging free-text reports (Figure 1).

**Figure 1:**
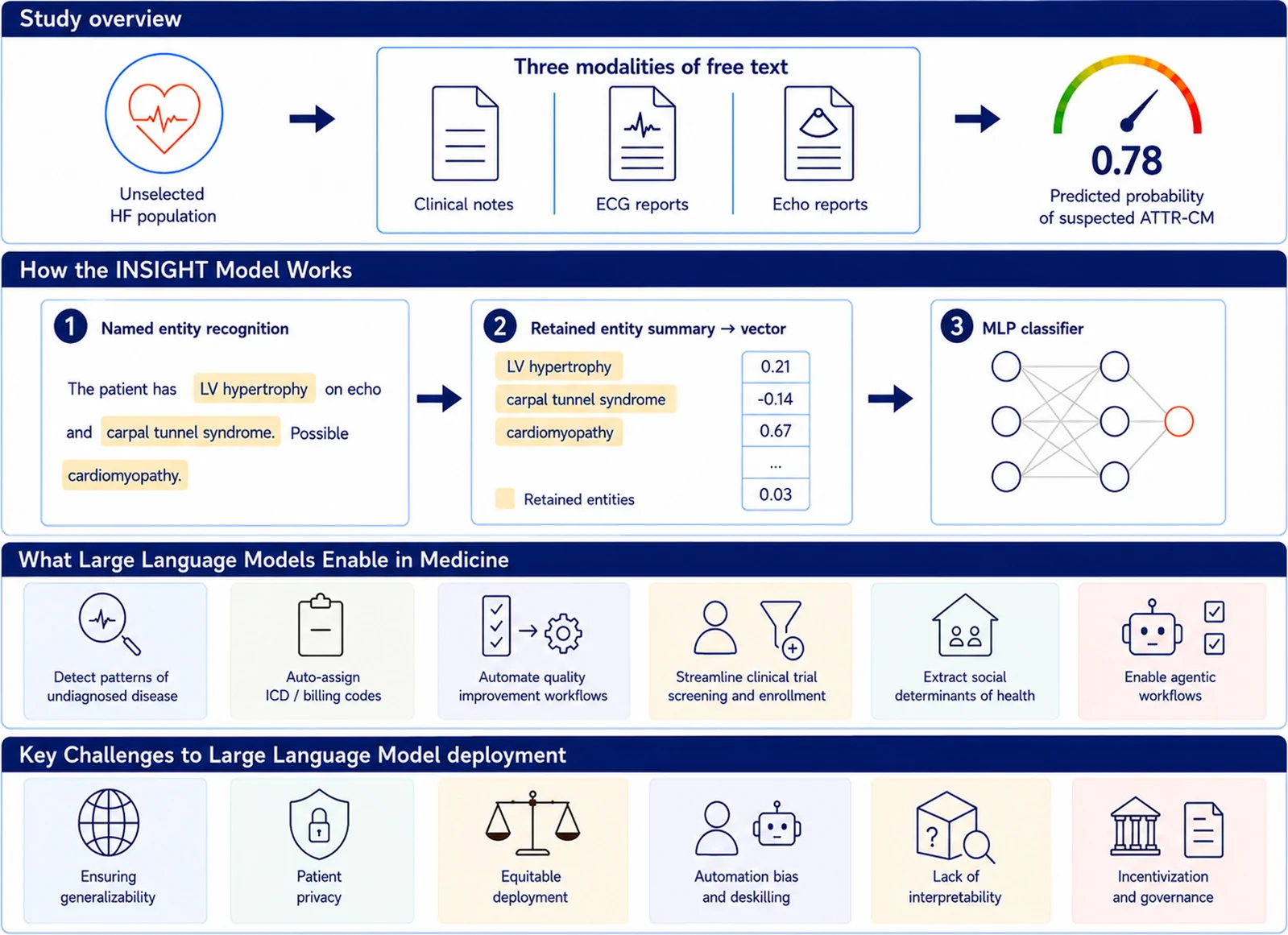
INSIGHT: Artificial Intelligence Screening for Suspected Transthyretin Amyloid Cardiomyopathy From EHR Text Top panel: Study overview. Second panel: INSIGHT model steps. Third panel: Overview of applications enabled by large language models in medicine. Bottom panel: Challenges to large language model deployment for disease screening. ATTR-CM = transthyretin amyloid cardiomyopathy; ECG = electrocardiography; EHR = electronic health record; HF = heart failure; LV = left ventricle; MLP = multilayer perceptron.

Briefly, the investigators developed and internally validated an NLP pipeline to identify patients with suspected ATTR-CM within an unselected heart failure population. Their data set comprised 100,290 patients with a heart failure diagnosis code from the Baylor Scott & White Health system in Texas, of whom 1.1% had an amyloidosis International Classification of Diseases (ICD) diagnosis code (E85.∗) and were classified as cases after light-chain amyloidosis (E85.81) was excluded. Cases were deliberately defined broadly when the E85 ICD-10-CM code was present to capture patients in whom clinical suspicion had been raised, rather than those with a confirmed disease, on the grounds that the intended output is a prompt for further evaluation. The pipeline had 3 stages. First, clinical notes, echocardiographic reports, and electrocardiographic narratives were passed through a named-entity recognition model fine-tuned on expert-annotated records, retaining recognized entities such as diseases, symptoms, echocardiographic and electrocardiographic findings, and their negations, while discarding the remaining text. Second, terms capable of revealing case status, including amyloid, ATTR, and pyrophosphate scan, were removed, and the resulting entity summaries were encoded by a second pretrained model into a fixed-length vector for each note. Third, these vectors were aggregated to the encounter level, with each patient represented by all encounters preceding the amyloidosis diagnosis date and passed to a multilayer perceptron classifier, with the patient as the unit of prediction. Structured data were used solely to construct the cohort and to report stratified performance and were not supplied as features.

On an internal held-out test set, the model achieved a sensitivity of 77.5%, specificity of 94.7%, and positive predictive value (PPV) of 19.9%, flagging 6.5% of the population as screen-positive. The authors note that INSIGHT achieves a higher PPV than previously reported screening approaches, including the Mayo Clinic Simple Score, which relies on structured clinical and echocardiographic variables and earlier NLP methods applied to unstructured data. Such comparisons across studies should be interpreted cautiously, as PPV depends heavily on cohort and case definitions, disease prevalence, and operating thresholds. Nonetheless, the emphasis on PPV is welcome and appropriate, as it is a metric that determines whether a screening tool can be embedded in a clinical workflow without generating an unmanageable burden of downstream testing.

Local Interpretable Model-agnostic Explanations were used to identify predictors most influential across the cohort. Those ranked highest were clinically coherent, including carpal tunnel syndrome, reduced global longitudinal strain, weight loss, and orthopnea, corresponding closely to recognized extracardiac and echocardiographic red flags. This concordance is reassuring, indicating that the model draws on established clinical signals rather than artifacts of institution-specific care practices. The clinical value of these explanations is also apparent, as a flag accompanied by the findings that generated it can be triaged by a clinician far more readily than a probability alone. However, two caveats to this methodology warrant mention. Because Local Interpretable Model-agnostic Explanations infer importance by testing how random variations in a patient’s recorded features change the prediction, the same patient can yield a somewhat different list on repeated runs, so the features shown are best read as an indication of what influenced a given flag rather than a fixed account of it. The cohort ranking, in turn, summarizes many such individual explanations and should be treated as descriptive rather than as a statement of which features the model relies on overall.

There are other important considerations in fully assessing the study's utility. The primary outcome of the study is a diagnosis code assignment, meaning that the model is developed and evaluated against contemporary practice rather than confirmed disease. The model learns to identify patients who resemble those in whom clinicians at one institution raised suspicion, potentially embedding local referral patterns alongside genuine disease features. It may thus assign weight to findings that clinicians believe indicate ATTR-CM but are not independently associated with it as such findings would precede a diagnosis code irrespective of their true predictive value. Future work evaluating the model in external patient cohorts who underwent confirmatory testing would help address this.

The necessity of the three-stage pipeline, which combines named-entity recognition, document embedding, and an multilayer perceptron classifier, is not firmly established. Of note, named-entity recognition extracts a set of clinical concepts, yet these features are never compared with structured features already recorded in the electronic health record. Nor is the pipeline compared with conceptually simpler alternatives. Encoding notes in full would dispense with the annotation step and retain more clinical context, while a pretrained generative model could be prompted to indicate directly whether a record raises suspicion of ATTR-CM, requiring neither an intermediate representation nor a trained classifier. Such approaches are increasingly accessible as capable open-source models become available for local deployment, and more complex pipelines should be justified by demonstrating improvements in performance, efficiency, or interpretability.

The path to implementation of such a model deserves specific discussion. During implementation, it is critical to define how a model of this kind would be embedded within a health system, which patients it should be run on, and how completion of downstream testing will be incentivized. Processing every note across a health system requires significant computational resources, so attention should be given to when and how often such models are run. A flag required in real time, at the point a patient is seen, imposes different demands from one generated periodically across a population and reviewed later. Whether inference runs within the record system itself or notes are passed to a separate environment is a further decision, with implications for cost, privacy, and latency. Indiscriminately running models on all heart failure patients and referring all individuals who flag positive for downstream testing may not be practical. Structured feature-based enrichment strategies that gate artificial intelligence model deployments can reduce computation workload and improve PPV while largely preserving sensitivity.^9^ Even with ideal model performance, prospective experience indicates considerable attrition between the patients a model identifies and those who complete diagnostic testing.^3^ Presenting model outputs as an electronic health record (EHR) embedded nudge is scalable and cheap but is readily disregarded, whereas a dedicated screening team may improve testing but is resource-intensive and risks concentrating benefit where detection is already best.^10^ Regulatory clearance has been granted to tools that screen for cardiac amyloidosis from single electrocardiograms and echocardiograms, but establishing an equivalent pathway for models that draw on the wider health record will be required for sustainable deployment.

In conclusion, Arora et al^8^ present important work developing and internally validating an NLP pipeline to identify patients with suspected ATTR-CM from unstructured clinical notes across an unselected heart failure population. These findings add to a growing literature demonstrating the utility of artificial intelligence applied to unstructured clinical documentation for automating the identification of patients with underrecognized conditions. Further work is needed to establish whether such pipelines identify disease rather than merely the suspicion of disease, whether their complexity is justified relative to simpler approaches, and how their outputs can be integrated into clinical workflows to better target diagnostic testing.

## Funding support and author disclosures

Dr Khera is an Associate Editor of *JAMA;* has received research support, through Yale, from the Blavatnik Foundation, Bristol-Myers Squibb, Novo Nordisk, and BridgeBio; is a co-inventor of U.S. Provisional Patent Applications 63/177,117, 63/428,569, 63/346,610, 63/484,426, 63/508,315, 63/580,137, 63/606,203, and 63/562,335; and is a co-founder of Ensight-AI, Inc. and Evidence2Health, LLC. All other authors have reported that they have no relationships relevant to the contents of this paper to disclose.
